# Ethnobotanical knowledge of *Astragalus* spp.: The world’s largest genus of vascular plants

**Published:** 2020

**Authors:** Mohammad Sadegh Amiri, Mohammad Reza Joharchi, Mohabat Nadaf, Yasamin Nasseh

**Affiliations:** 1 *Department of Biology, Payame Noor University, Tehran, Iran*; 2 *Department of Botany, Research Center for Plant Sciences, Ferdowsi University of Mashhad, Mashhad, Iran*

**Keywords:** Astragalus, Ethnobotany, Fabaceae, Vascular plants, World

## Abstract

**Objective:**

*Astragalus* L. (Fabaceae) is the largest genus of vascular plants in the world, that comprises an estimated number of 2900 annual and perennial species. The members of this genus have a broad spectrum of usages (e.g. medicine, food, fodder, fuel, ornamental plants, etc.). Here, we present a review of ethnobotanical applications of different species of *Astragalus *by various ethnic and cultural groupings worldwide, to provide an exhaustive database for future works.

**Materials and Methods:**

Literature survey was performed using Scopus, Google Scholar, PubMed, Medline, and Science Direct, and English and non-English reference books dealing with useful properties of the *Astragalus *species from 1937 to 2018. Consequently, we reviewed a total of 76 publications that supported lucrative information about various uses of this huge genus.

**Results:**

Several ethnobotanical uses of 90 *Astragalus *taxa were documented which were mainly originated from Asian and European countries. The two most frequently mentioned *Astragalus *treatments, were against urinary and respiratory diseases. The most commonly used part was gum and the most frequently used preparation method was decoction.

**Conclusion:**

This review highlights that various *Astragalus* species have great traditional uses in different ethnobotanical practices throughout the world. However, there is still lack of phytochemical and pharmacological researches on many species of *Astragalus* and further studies are required to substantiate the therapeutic potential of them which will develop new generation of plant-derived drugs in the near future.

## Introduction

The genus *Astragalus *L. belongs to the well-known plant family Fabaceae and tribe Galegeae, which has high medicinal and economic values. The genus *Astragalus *is the largest genus of vascular plants with approximately 2900 species, which has two main centers of distribution in the world, America (New World) and Eurasia (Old World). Most of the species are located in the Old world (ca. 2400 spp.) whereas ca. 500 species are restricted to the New World (Chaudhary et al., 2008 ▶, Zarre and Azani, 2013 ▶). It is a considerable example of adaptive radiation in a worldwide scale (Kazempour Osaloo et al., 2003 ▶). From a biogeographic point of view, *Astragalus* is a characteristic Irano-Turanian element and many species of it show a narrow geographic range (narrow endemics), which makes them particularly vulnerable to extinction (Jalili and Jamzad, 1999 ▶; Memariani et al., 2016 ▶). Iran is known for its high diversity of *Astragalus *that comprises 850 species of *Astragalus*, of which 527 species are endemics (Maassoumi, 1998 ▶; Maassoumi, 2005 ▶). Morphologically, its members can be broadly characterized by the presence of typical papilionaceous flowers. *Astragalus *species differ from short living annual herbs (ca. 80 spp.) to perennial rhizomatous or hemicryptophytic herbs (ca. 2500 spp.) and to cushion forming spiny shrubs (ca. 300 species). Most members of the genus are generally associated with semi-arid and arid habitats across the world, however, a few species select humid habitats (e.g. *A. glycyphyllos *L.), or are known as weeds. Due to the large size of the genus, it has fascinated different investigators, but much work remains to be done. There is much confusion regarding *Astragalus *taxonomy and phylogeny. Several authors have attempted to subdivide *Astragalus* to achieve a natural subgeneric classification by means of morphological characters. Among them, Bunge’s classification of the genus (eight subgenera and 105 sections), has been extensively employed until recently (Zarre and Azani, 2013 ▶; Maassoumi, 1998 ▶; Maassoumi, 2005 ▶). As the largest genus of vascular plants, its circumscription will remain obscure until the majority of known morphological lineages, are surveyed for adequate numbers of plastid and nuclear markers (Zarre and Azani, 2013 ▶).

In the literature, multiple reports have described various ethnobotanical aspects of different species of the genus *Astragalus*. These invaluable plants are widely used as medicine, food, fodder, fuel and as ornamental plants in different ethnobotanical practices throughout the world (Table1). The most used part of *Astragalus* taxa is the gum tragacanth and Iran is the primary source of it (by supplying 70% of the commercially used gum tragacanth) in the world (Anderson and Grant, 1988; Anderson, 1989). Nowadays, several species of *Astragalus*, are reported to be commercially exploited for gum tragacanth (Table2). Despite the vast ethnobotanical knowledge on this genus that exists around the world, there are no distinct references on its applications and most of the publications are widely scattered. Furthermore, the number of phytochemical and pharmacological studies conducted on this big genus, is still too few. Therefore, this review aims to integrate the findings concerning the ethnobotanical aspects of *Astragalus* genus in order to support sufficient baseline data for subsequent works and commercial exploitation.

## Materials and Methods

This review was prepared based on an extensive survey of major scientific databases namely, Google Scholar, Scopus, PubMed, Medline, and Science Direct, and English and non-English reference books dealing with useful properties of the *Astragalus *species over the past few decades (1937- 2018). After a holistic search, we reviewed a total of 76 publications that reported beneficial information about various aspects of the genus *Astragalus* globally. The most frequently published reports on this genus were from various regions of Iran, Turkey, India, Pakistan, China and American countries. In this paper, scientific and author names of plant species were carefully scrutinized for latest changes via “The Plant List” (http://www.theplantlist.org) and also according to the most recent monograph of the genus (Podlech and Zarre, 2013 ▶).

## Results

**Figure 1:**
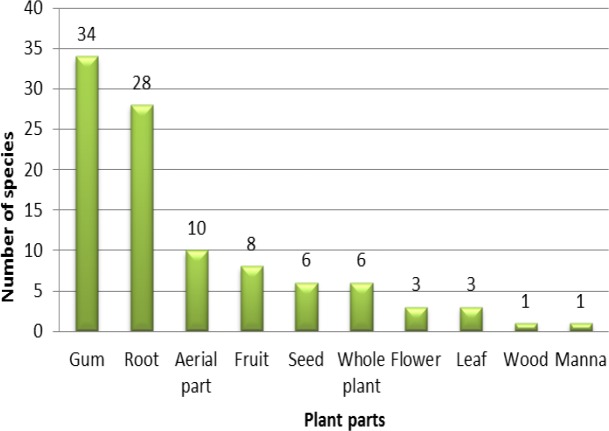
Proportional presentation of most used plant parts

In this review, several ethnobotanical usages of 90 *Astragalus *taxa which mainly originated from Asia and Europe, were documented. These invaluable plants were arranged in alphabetical order for their scientific names, with the related data. The information comprises autochthonous names, the part(s) used, the method of preparation, and traditional applications along with literature sources. Various parts of *Astragalus* taxa have been used in different ethnobotanical practices around the world. The most used parts of the plants were gum (34 species) followed by root (28 species), aerial part (10 species), fruit (8 species), seed (6 species), whole plant (6 species), flower (3 species), leaf (3 species), wood (1 species) and manna (1 species) (Figure 1). 

**Figure 2:**
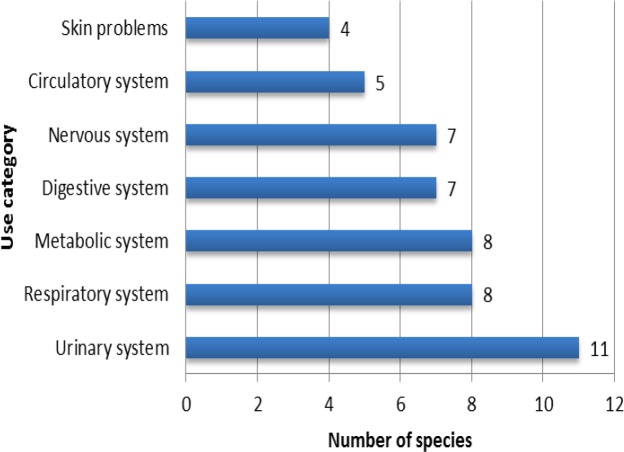
Proportional presentation of species applied in each medicinal use category

The most common methods of preparation were decoction (20 species), followed by infusion (5 species), poultice (4 species), chewing (4 species), powder (4 species) and bath (1 species). Many reports could be found representing ethnomedicinal uses of different members of this huge genus. Among them, *A. brachycalyx* Fisch. ex Boiss. (Syn.* A. adscendens* Boiss. & Hausskn.), *A. fasciculifolius* Boiss., *A. glycyphyllos* L., *A. gossypinus* Fisch., *A. gummifer* Labill., *A. hamosus* L., *A. microcephalus* Willd., *A. mongholicus *Bunge and *A. tribuloides* Delile are the most popular medicinal plants. The most treated illness categories were the urinary system (11 species), respiratory system (8 species), metabolic system (8 species), digestive system (7 species), nervous system (7 species), blood and circulatory system (5 species), and skin problems (4 species) (Figure 2).

**The importance of ethnobotanical aspects**

Literature review indicated that many cultures including Asian, European, American and African have used *Astragalus* species for alleviating a wide array of diseases. Some of its exemplary uses are given below while the others are summarized in Table 1. In Asian countries, particularly Iran, Pakistan, India, China and Korea, there are considerable reports on the traditional applications of *Astragalus* species. Iran is a region of high *Astragalus *biodiversity and thus, the rich tradition usage of *Astragalus *in this area is not surprising. Among them, *A. brachycalyx* Fisch. ex Boiss.*, A. fasciculifolius* Boiss.*, A. fischeri *Buhse ex Fisch.*, A. globiflorus* Boiss.*, A.gossypinus* Fisch.*, A. gummifer* Labill.*, A. hamosus* L.*, A. mucronifolius* Boiss., *A. ovinus* Boiss. and* A. verus* Olivier*, *are the most commonly used ones in different regions of Iran. The common Persian name of the majority of *Astragalus *species is “Gavan” (Mozaffarian, 2007 ▶). In Iran, the decoction of aerial parts of *A. hamosus* L. is considered very useful in the treatment of prostate problems. The decoction of flower, root and gum of *A. fasciculifolius* Boiss. locally known as “Gineh or Ginja”, is recommended for the treatment of cold, joint pains, aching tooth, wounds and diabetic wounds (Mosaddegh et al., 2012 ▶). The decoction of its gum popularly known as “Anzerut”, is also broadly used in Iranian Traditional Medicine system, as antitussive, laxative, and anthelmintic and to cure jaundice (Amiri et al., 2014 ▶; Mozaffarian, 2013 ▶). Moreover, it is used as a remedy for cold, and fatigue and for tightening bone fractures. Root decoction of* A. mucronifolius *Boiss. is also considered very useful in the treatment of back pain and bone fracture by traditional healers of Iran (Safa et al., 2013 ▶). In Traditional Chinese Medicine (TCM),* A. mongholicus *Bunge (Syn.* A. membranaceus* (Fisch.) Bunge;* A. propinquus* Schischkin), is one of the most famous tonic herbs. It is also an antiperspirant, and a diuretic, and is consumed for treatment of nephritis and diabetes (Yu et al., 2013 ▶). In Pakistan, the roots of *A. mongholicus *Bunge commonly known as “Shatra”, are widely used in traditional medicine as an adaptogenic, immune stimulant, diuretic, vasodilator and antiviral agent (Ullah et al., 2014 ▶). In Jordan, the fruits of *A. hamosus* L. are applied externally as incense, and evil eye and for treatment of baldness (Lev and Amar, 2002 ▶).

The genus *Astragalus* is also well documented for its notable applications in the European Traditional Medicine. In Russian folk medicine, *A. laxmannii *Jacq. (Syn. A. adsurgens Pall.), A. dahuricus (Pall.) DC. and A. penduliflorus Lam. are used as a diuretic for treatment of oedema. In Belarus, A. arenarius L. and A. cicer L. are applied to heal heart and gastrointestinal diseases. In Bulgarian folk medicine, A. corniculatus M. Bieb., A. ponticus Pall. and A. vesicarius L. are used as a diuretic for treatment of hypertension, renal system ailments, nervous disorders and rheumatism**, **and as a diaphoretic (Lysiuk and Darmohray, 2016 ▶). Furthermore, a decoction of root of *A. gummifer* Labill. is used for diabetes (Çakılcıoğlu et al., 2010 ▶). Some other *Astragalus* species are also well documented for their folkloric use as anti-diabetics in traditional medicine of Turkey, Lebanon and Iran (Table1). In different geographical areas of American continent, remarkable reports that highlight ethnobotanical and traditional applications of the genus *Astragalus*, are found. In Argentina, A. mongholicus Bunge is used as an antifatigue, antistress (adaptogenic), antiaging, neuroprotective, and cognitive enhancer agent, and to treat sexual dysfunctions and genital sickness (Hurrell and Puentes, 2017 ▶). In the USA, various species of *Astragalus *such as *A. americanus* (Hook.) M. E. Jones, *A. amphioxys* A. Gray, A. canadensis L. and A. crassicarpus Nutt., are applied to treat different ailments (Table1). In African continent, the root and seed of *A. armatus* Willd. are used traditionally in the Algerian folk medicine as an effective treatment for leishmaniasis and helminthiasis (Chermat and Gharzouli, 2015 ▶). Moreover, *A. arpilobus *subsp. *hauarensis *(Boiss.) Podlech (Syn. *A. gyzensis* Bunge), called “Foul Alibil”, is used against scorpion stings and snake bites (Lakhdari et al., 2016 ▶). In Ethiopia, the fresh chewing and poultice of A. atropilosulus (Hochst.) Bunge leaf known as “Teten agazen”, is applied to treat teeth pain (Hishe and Asfaw, 2014 ▶). *Astragalus *taxa are reportedly used for a multitude of ethnobotanical purposes besides medicine consumption.

**Table 1:** Importance of ethnobotanical applications of *Astragalus* taxa in different countries around the world

**Reference cited**	**Ethnobotanical uses**	**Preparation**	**Parts used**	**Vernacular name**	**Country**	**Scientific name**	**NO**
	Kidney disease, hypertension, burns, demulcent	-	Leaves, gummy exudation	Astragal	Uzbekistan	*A. abolinii* Popov	1
	Stomach pain and flu	Chewing	Root	American milkvetch	America	*A. americanus* (Hook.) M.E.Jones	2
	Galactagogue in animals	-	Whole plant	Oaxxai	Pakistan	*A. amherstianus* Benth.	3
	Rattlesnake bite	The root is chewed by the medicine man before sucking upon the wound; chewed root is applied to the bite	Root	Chitdola awan ak'wa	America	*A* *.* *amphioxys* A.Gray	4
	Ornamental, medicinal plant used as astringent	-	Root	Kotad Haramon	Lebanon	*A. angustifolius* Lam.	5
	It used as animal fodder	-	Root	Kokar geven	Turkey		
	leishmaniasis , helminthiasis	-	Root, seed	-	Algeria	*A. armatus* Willd.	6
	It used as animal fodder	-	Whole plant	Geven, Eşşek geveni	Turkey	*A. baibutensis* Bunge	7
	Laxative, febrifuge, and digestive	-	Manna	Gazangabin	Iran	*A. brachycalyx* Fisch. ex Boiss. (Syn.* A. adscendens* Boiss. & Hausskn.)	8
	Diabetes	Decoction	Root	Gewen	Turkey		
	Crushed roots are applied as animal fodder	-	Root	Geven	Turkey	*A. cadmicus* Boiss.	9
	Analgesic, eaten raw or boiled in blood to make broth	-	Root	Canadian milkvetch	America	A. canadensis L.	10
	Roots are pounded to obtain a gum as glue. In winters spiny leaves of the plant are pounded after moistened with water and used as animal fodder	-	Whole plant	Keven	Turkey	*A. compactus* Lam.	11
	Cold	Decoction	Seed	Eklilolmolk	Iran	*A. camptoceras* Bunge	12
	Taken orally to treat diabetes and jaundice	Decoction	Root	Kitad Kansouri	Lebanon	*A. coluteoides* Willd.	13
	Root decoction is tonic, anticonvulsive and anti-headache, fruits are eaten raw as a snack, pods are consumed raw, cooked, or pickled	Decoction, eaten raw	Root, fruit, pods	Groundplum milkvetch	America	A. crassicarpus Nutt.	14
	Kidney stone, sedative, arthrodynia, carminative	-	Fruit	Nakhonak	Iran	*A. crenatus* Schult. (Syn. A. corrugatus Bertol.)	15
	Used as sedative and tonic	-	Aerial parts	Aghazi Shatra	Pakistan	*A. creticus* Lam.	16
	Ornamental plant, taken orally to treat diabetes and jaundice	Decoction	Root	Kotad Ahmar	Lebanon	*A. cruentiflorus* Boiss.	17
	Eaten raw as a snack	Raw	Unripe seeds	Cornizuelos	Spain	*A. cymbicarpos* Brot.	18
	Making tragacanth, used as detergent, Produce rope	-	Gum	Gini	Iran	*A. dschuparensis* Freyn & Bornm.	19
	Cough	Boiled, brewed	Gum	Gavan	Iran	*A. effusus* Bunge	20
	Tightening the roots of teeth, cough, nutritious, kidney, stomach ache, chest infection, toothache	Decoction, infusion, poultice	Stem, seed, root	Gonjed	Iran	*A. fasciculifolius* Boiss.	21
	Toothache, back ache, bone ache, kidney ache, bone fracture, and diabetes, and to induce abortion	Decoction, raw, poultice	Aerial parts, seed, root	Shoun korouchok	Iran	*A. fischeri *Buhse ex Fisch. (Syn.* A. phyllokentrus *Hausskn. & Bornm.)	22
	Used in food and confectionery, tonic, gastric pain, headache	-	Fruit	Miveh badkonaki	Iran	A. glaucacanthos Fisch.	23
	Healing deep infectious wounds	Powdered gum with Teucrium	Gum	Gineye gaamur	Iran	*A. globiflorus* Boiss.	24
	Increasing men’s sexual potency	-	Aerial parts	Orlovi nokti	Montenegro	*A. glycyphyllos* L.	25
	The roots and leaves are used for their refreshing, purifying, and diuretic properties. They were also used for kidney ailments, gout and rheumatism.	-	Root, leaves	Astragalo	Italy		
	Cough	Boiled, brewed, incense	Gum	Gavan panbei	Iran	*A. gossypinus* Fisch.	26
	Used for treatment of abscesses and as an analgesic	-	Whole plant	Aghazi Shatra	Pakistan	A. grahamianus Benth.	27
	Diabetes	Infusion	Root	Günizer	Turkey	*A. gummifer* Labill.	28
	The decoction of the beans is used internally in nervous system disorders; liver, kidney and spleen infection. The paste of the beans is massaged and applied externally on inflamed areas	Decoction, paste	Fruit	Iklilul Malik, Nakhuna	India	*A. hamosus* L.	29
	Powdered seeds and flowers given in strangury	Powder	Flower, Seed	Kayabachtp	India	A. himalayanus Klotzsch	30
	Typhoid, and dermal problems	Decoction, bath	Root	Haram-chop	Iran	*A. jolderensis* B.Fedtsch.	31
	Ulcer	Decoction	Root	Cuni	Turkey	*A. lamarckii* Boiss.	32
	Used as fuel wood	-	Aerial parts	Kathi	Pakistan	A. leucocephalus Bunge	33
	Cardiac disorder, diabetes	Infusion	Root	Gırguni	Turkey	*A. longifolius* Lam.	34
	Asthma, strengthen hair	Infusion, pulverized	Stem, root	Kalelak	Iran	*A. microcephalus* Willd.	35
	Against body weakness, diuretic, against digestive system disorder, as supplement in cosmetic. Stem and leaves used as animal feed	-	Root	Huangqi	China	*A. * *mongholicus* Bunge (Syn. *A. membranaceus* (Fisch.) Bunge;* A. propinquus* Schischkin )	36
	Hypertension, dyspepsia, and common cold	**-**	Root	Astragalos	Greece		
	Blood circulation	Decoction	Root	Hwanggi	Korea		
	Diuretic	-	Root	Astragalo rosato	Italy	*A.* *monspessulanus* L.	37
	Backache	-	Root	Gonjar khari	Iran	*A. mucronifolius* Boiss.	38
	Varicosis	Crushed	Root	Keven	Turkey	*A. noaeanus* Boiss.	39
	Ornamental plant, decoction of roots is orally applied as an emollient and as a remedy for diabetes and jaundice	Decoction	Root	Kotad Jareh	Lebanon	*A. oleifolius* DC.	40
	Nutritious	Raw	Aerial parts, fruit	Kahour kah	Iran	A. ophiocarpus Boiss.	41
	Shoots are collected in summer, stored and used as fuel wood in winter	-	Shoot	Dume ruba	Pakistan	*A. oplites* Benth. ex R. Parker	42
	Stomachache,used in pickle	Orally	Fruit	Gondkhorosi	Iran	*A. ovinus* Boiss.	43
	Bellyache, and colic	Decoction, raw	Aerial parts, leaf, flower	Katek	Iran	A. podolobus Boiss. & Hohen.	44
	Cataract, and stomach problems	-	Aerial parts	Maakhai	Pakistan	*A. psilocentros* Fisch.	45
	It is used as animal fodder	-	Root	Zomoshing	India	*A. rhizanthus* Benth.	46
	Digestive disorders, Leucorrhea, and urinary troubles	-	Root	Rudravanti	India	*A. * *rhizanthus *subsp.* candolleanus* (Benth.) Podlech (Basionym: *A. candolleanus* Benth.)	47
	Kidney disease, hypertonic disease, burns, demulcent	-	Leaves, gummy exudation	Astragal	Uzbekistan	A. rubrivenosus Gontsch.	48
	Incense, pains	-	Gum	Sarcocolla	Jordan	*A. sarcocolla* Dymock	49
	Menstrual Disorders	-	Fruit	Gol Sefid	Iran	*A. sieversianus* Pall.	50
	Gastric troubles, swelling and joint pains	Powder	Whole plant	Satkar	India	*A. thomsonianus* Benth. ex Bunge	51
	Toothache	Chewing	Gum	Geven, Saçaklı geven	Turkey	*A. tmoleus* Boiss. (Syn.* A. tmoleus* subsp. *bounacanthus* (Boiss.) Ponert )	52
	Whole plant is applied as a diuretic agent and to lower kidney disorders. Root extract purifies blood.	-	Whole plant	Yanglo	India	*A. tribulifolius* Bunge	53
	Urinary infection	Infusion	Arial part	Sareng, Sateng	Iran	*A. tribuloides* Delile	54
	Antiparasitic, antimycotic and immunomodulatory activities	-	Wood	Siahgavan	Iran	*A. verus* Olivier	55
	Against worms	-	Arial part	-	India	A. zanskarensis Bunge	56

 In Iran, the manna of *A. brachycalyx* Fisch. ex Boiss. is applied in preparation of honey and traditional Iranian sweet candy (“*Gaz*” in Persian) (Golmohammadi, 2013 ▶). In Turkey, the root of *A. condensatus *Ledeb. (Syn. *A. brachypterus* Fisch.) and *A. microcephalus* Willd. are pounded to obtain gum which is used as glue (Özüdoğru et al., 2013 ▶). Several species of *Astragalus *such as A. alpinus L. (bluish-purple flowers), A. hypoglottis L. (purple flowers), and *A. sinicus* L. (Syn. *A. lotoides* Pall.), are grown as ornamental plants in gardens (Golmohammadi, 2013 ▶). Some species of *Astragalus *including and *A. oplites* Benth. ex R. Parker are collected in summer, stored and used as fuel wood in winter (Ali and Qaiser, 2009 ▶; Hussain and Muhammad, 2009 ▶). Furthermore, some plants like *A. angustifolius* Lam.*, A.* *baibutensis* Bunge*, A. cadmicus* Boiss., *A. compactus* Lam.*, A. mongholicus *Bunge and* A. rhizanthus* Benth. were documented as forage for livestock (Table1). 

**Tragacanthic species of** ***Astragalus***

Several tragacanthic species of the genus *Astragalus* gained fame owing to their potential in producing gum tragacanth which has a wide array of uses in medicine and many industries. Among them, *A. gummifer* Labill., *A. microcephalus* Willd., *A. brachycalyx* Fisch. ex Boiss., *A. myriacanthus* Boiss. (Syn. *A. echidnaeformis* Sirj.), *A. gossypinus* Fisch. and *A. kurdicus* Boiss. are the most important species to supply the gum tragacanth in global market (Verbeken et al., 2003 ▶). However, the contribution of other tragacanthic species is also significant (Table 2). The name "tragacanth" is derived from the two Greek words tragos (goat) and akantha (horn), referring to the white curled ribbons, the best grade of commercial gum (Whistler, 1993 ▶). Gum tragacanthic plants are perennial legumes, characterized by spine-tipped leaf rachises; sessile or subsessile flowers, glomerate in the axils of the leaves; and one-seeded pods enclosed in hairy persistent calyces (Gentry, 1957 ▶). Iran is well-known as the largest producer and exporter of gum tragacanth and supplies the highest quality of it for the world (Anderson and Grant 1988 ▶). Turkey is the second largest producer, but Turkish gum is deemed to be of an inferior quality. Much smaller amounts of gum are exported by Afghanistan and Syria (Verbeken et al., 2003 ▶). The United States, the United Kingdom, Russia, Germany, France, Italy and Japan have been the biggest importers of gum tragacanth (Whistler, 1993 ▶). Structurally, gum tragacanth is categorized into two general kinds, ribbon (highest grade) and flake or “kharmony”. After collection, Iranian tragacanth ribbons are classified into five grades, while flakes are sold in seven various grades (Gentry, 1957 ▶; Verbeken et al., 2003 ▶). 

Gum tragacanth comprises of two fractions including Tragacanthin (water-soluble) and Tragacanthic acid or bassorin (water-insoluble). Although the latter is insoluble in water, but has the capacity to swell and form a gel (Anderson and Bridgeman, 1985 ▶). Commercially, gum tragacanth has extensive applications as an emulsifier, stabilizer and thickening agent in various industries, due to its stability to heat and acids and because it is an effective emulsifying agent with an extremely long shelf life (Whistler, 1993 ▶). However, there are several reports in the literature that compositional discrepancies of gum tragacanth obtained from diverse tragacanthic species of *Astragalus*, can result in the chemical and physical changes (Balaghi et al., 2011 ▶). 

**Therapeutic and pharmaceutical applications**

Gum tragacanth has been used therapeutically for thousands of years, with written evidence of its applications, described by Theophrastus in the 3rd century B.C. (Whistler, 1993 ▶). In some Asian countries, particularly Iran, various tragacanthic species of *Astragalus *have a broad habitation and many of them are important in folk medicine (Table2). In Iran, the tragacanth gum, commonly known as “*Katira*’, has been largely used in medicine and confectionery since ancient times (Hopper and Field, 1937 ▶). In Iranian traditional medicine, gum tragacanth is broadly applied as an analgesic, general tonic, and laxative agent and to cure cough and lip fissures (Zarshenas et al., 2013 ▶). In Jordan, the gum of *A. gummifer* Labill., commonly known as “Tragacanth’, is widely employed in traditional medicine for healing stomachache and coughs (Lev and Amar, 2002 ▶). 

In addition to its usage in traditional therapeutics, the gum tragacanth has also been applied as an excellent suspending agent for many pharmaceutical products. Mucilage of tragacanth is utilized in lotions for external applications. It is also applied at higher concentrations as a base for jelly lubricants. Gum tragacanth can act as the suspending agent in various kinds of toothpastes with a humectant, such as glycerol or propylene glycol. It forms a creamy and brilliant product. Its long shelf life and its film-forming properties make it beneficial in hair lotions and hand creams and lotions (Whistler, 1993 ▶).

**Table 2:** Some of the most important tragacanthic species of *Astragalus *L. (Fabaceae).

**NO**	**Scientific name**	**Reference **
1	*A. albispinus* Sirj. & Bornm.	
2	*A. andalanicus* Boiss. & Hausskn.	
3	*A. brachycalyx* Fisch. ex Boiss. (Syn. *A. adscendens* Boiss. & Hausskn.; A. leioclados Boiss.)	Gentry, 1957 ▶; Mozaffarian, 2013 ▶
4	*A. * *brachycalyx *subsp. *eriostylus *(Boiss. & Hausskn.) Zarre (Basionym: *A. eriostylus* Boiss. & Hausskn.)	
5	*A. caspicus* M.Bieb.	
6	A. cerasocrenus Bunge	
7	*A. compactus* Lam.	
8	*A. * *condensatus *Ledeb. (Syn. *A. brachypterus* Fisch.)	
9	*A. creticus* Lam.	
10	*A. cylleneus* Boiss. & Heldr. ex Fischer	
11	*A.* * cymbostegis *Bunge (Syn. *A. stromatodes *Bunge)	
12	*A.* * diphtherites *Fenzl (Syn. *A. strobiliferus *Benth.)	
13	*A. dschuparensis* Freyn & Bornm.	
14	*A. echidna* Bunge	
15	*A. * *eriosphaerus *Boiss. & Hausskn. (Syn. *A. elymaiticus *Boiss. & Hausskn.)	
16	*A. floccosus* Boiss.	
17	*A. * *floccosus *subsp. *rahensis *(Širj. & Rech.) Zarre (Basionym: *A. rahensis* Sirj. & Rech.f.)	
18	*A. geminanus* Boiss. & Hausskn.	
19	*A. globiflorus* Boiss.	
20	*A. gossypinus* Fisch.	
21	*A. hypsogeton* Bunge	
22	*A. kurdicus* Boiss.	
23	*A. longistylus* Bunge	
24	*A. microcephalus* Willd. (Syn. *A. senganensis *Bunge)	Mozaffarian, 2013 ▶; Gentry, 1957 ▶
25	*A. * *muschianus *Kotschy & Boiss. (Syn. *A. gummifer* Labill.)	
26	*A. myriacanthus* Boiss. (Syn. *A. echidnaeformis* Sirj.)	
27	*A.* * microcephalus *subsp*. pycnocladus *(Boiss. & Hausskn.) Širj. (Basionym: *A. pycnocladus *Boiss. & Hausskn.)	
28	A. pycnocephalus Fisch.	
29	A. tragacantha L.	
30	*A. verus* Olivier (Syn. *A. *brachycentrus Fisch.;* A. heratensis* Bunge;* A. meschhedensis* Bunge;* A. parrowianus* Boiss. & Hausskn.)	Mozaffarian, 2013 ▶; Gentry, 1957 ▶; Gavlighi et al., 2013 ▶

**Food applications**

Due to its acid resistance and its long shelf life, gum tragacanth is lucrative in the preparation of different kinds of salad dressings, relishes, sauces, condiment bases, sweet pickle liquors, soft jellied products such as gefilte fish, thick broths, beverage and bakery emulsions, ice cream and sherbets, bakery toppings and fillings and confectionary (Whistler, 1993 ▶).

**Miscellaneous applications**

Gum tragacanth can be employed in various kinds of polishes for furniture, floor, and auto polishes. It is beneficial in print pastes and sizes because of its good release properties. The gum is applied for stiffening silks and crepes. It is also utilized in the dressing of leather and in the preparation of leather polishes. Certain grades of gum tragacanth are lucrative as binding agents in ceramics because they contain a low ash content, and the gum acts to suspend different materials in a mass prior to the firing of the ceramic in the furnace. Gum tragacanth forms stable emulsions containing 50% insect repellant. They have the potential to be efficacious as pure repellant compounds against mosquitoes, mites, chiggers, ants, and certain fleas (Whistler, 1993 ▶).

**The importance of identification credibility in ethnobotany**

The validity of botanical identification is the first step in the ethnobotany studies. Nowadays, the ethnobotanical investigations can comprise a few erroneous and ambiguous identifications, due to the lack of services of taxonomic or botanical expertise. For instance, in the present literature review, we found that the gum of *A. ammodendron* Bunge has been employed for ethnobotanical applications (Sadeghi et al., 2014 ▶). However, this taxon does not occur in Iran (Podlech and Zarre, 2013 ▶). Thus, we omitted this plant from the list (Table 1). Moreover, one of the major problems ethnobotanists face is when identical names are attributed to various species, or different names to the same species. For example, the name Eklilolmolk is matched with *A. camptoceras* Bunge and *Melilotus officinalis* (L.) Pall. in different references (Rajaei et al., 2012 ▶; Mozaffarian, 2007 ▶). Therefore, correct identification of plant species should only be authenticated by a panel of experts including taxonomists (Joharchi and Amiri, 2012 ▶). Additionally, preparation of voucher specimens are crucial for scientific identification which can diminish such mistakes and help investigators with a better perception of their subjects (Bennett and Balick, 2008 ▶). 

## Discussion

The genus *Astragalus *is one of the most important genera in the Fabaceae family. This review highlights that different *Astragalus* species have great potential uses in medicine and many industries. The total number of 90 *Astragalus* taxa (the sum of the species in Table 1 and 2, as well as the taxa pointed in the text without repetition), reveals numerous ethnobotanical and ethnomedicinal applications around the world. Ethnomedicinal results showed that the most frequent traditional applications of *Astragalus* taxa in different countries, seems to be treatment of urinary diseases, respiratory ailments, digestive diseases*, *nervous ailments, circulatory diseases, skin problems, and as an antiseptic, tonic and antidiabetic agent. The most used part of *Astragalus* taxa is gum which has a broad applications in different industries, due to its outstanding characteristics. However, it is important to note that the gum exudate from various tragacanthic species of *Astragalus *has diverse chemical composition, so exhibits different properties and behaviors. Hence, any try for development of biomedical uses of gum tragacanth without considering the plant species, will result in misleading information (Balaghi et al., 2011 ▶). To our knowledge, there is still lack of phytochemical and pharmacological researches on many species of *Astragalus* that have been traditionally used in various countries. In addition, there are only few reports on the biological activity of some *Astragalus *species, of which the majority have investigated the anticancer effects (Yesilada et al., 2005 ▶). In this context, the best and quickest way to species selection for phytochemical, biological and pharmacological studies, is by reviewing the ethnobotanical literature which highlights the importance of such studies (Amiri and Joharchi, 2016 ▶). Based on the data presented in this paper, some species should be given precedence to subsequent investigations, particularly, for the treatment of some globally prevalent diseases like diabetes including, *A. brachycalyx* Fisch. ex Boiss.,* A. coluteoides* Willd*., A. cruentiflorus* Boiss*., A. fischeri *Buhse ex Fisch*., A. gummifer* Labill.,* A. longifolius* Lam. and *A. oleifolius* DC. (Table 1). Besides the detailed information introduced in this article, supporting pharmaceutical and clinical trials should be undertaken to validate the therapeutic potential of different species of *Astragalus* which will develop new generation of herbal-based natural drugs for the optimal benefit to mankind. Finally, several* Astragalus *species, display a narrow geographic range (narrow endemics), with significant commercial and therapeutic value. These species are threatened due to intense harvesting pressure from the wild. Undertaking ecological investigation in *Astragalus *diversity hotspots such as Iran and Turkey is essential to conservation of this invaluable genus in these regions. 
